# Optimization of Steel Roof Framing Taking into Account the Random Nature of Design Parameters

**DOI:** 10.3390/ma15145017

**Published:** 2022-07-19

**Authors:** Paweł Zabojszcza, Urszula Radoń

**Affiliations:** Faculty of Civil Engineering and Architecture, Kielce University of Technology, 25-314 Kielce, Poland; zmbur@tu.kielce.pl

**Keywords:** steel roof framing, stability, reliability, deterministic optimization, robust optimization

## Abstract

The main subject of this paper is an optimization of steel roof framing used as a load-bearing structure in commercial pavilions. The authors wanted to draw attention to the necessity to take into account the uncertainty in the description of design parameters during optimization. In the first step, using geometrically nonlinear relationships, a static-strength analysis is performed. The decisive form of loss of stability in this steel roof framing is the jump of the node (snap-through), and not the buckling of the most stressed structure bars. Therefore, when creating the limit function, it was decided to make a condition limiting the permissible displacement. Values of the implicit limit function were calculated with Abaqus software based on the finite element method. Reliability analysis, and robust and deterministic optimization were performed using Numpress Explore software. Numpress Explore software communicates with the Abaqus software to perform analysis. The task ended with the generation of information that contained the failure probability, reliability index and the values of optimized areas of the bars’ cross-sections. The end result of the optimization is not a cost analysis, but an assessment of the safety of the structure.

## 1. Introduction

Steel roof frames and trusses are used as load-bearing structures in industrial halls, commercial pavilions, station concourses, sports halls, etc. In other words, they are used where a large clear space between columns is required [1].

The structures analyzed in this paper are often made of class 1 steel bars in which, after checking the strength of the bar cross-section, its overall stability should be additionally assessed. The overall stability of the members is assessed by verifying the resistance of members that are simultaneously in compression and bending. The general instability of a compression element, called buckling, can take one of three forms: bending (the element is bent in the plane of the lowest stiffness); torsional (the element twists around its longitudinal axis); and bending-torsion (the element is bent while twisting). The general instability of the member under a bending moment, called torsional buckling, occurs when the bending moment reaches a critical value. The buckling of the beam is initiated by the buckling of the compressed flange from the bending plane and immediately transforms into the beam torsion. The slenderness of a beam determines its loss of stability. With a suitably small slenderness, the beam is stable. Its susceptibility to loss of overall stability increases with increasing beam slenderness. The verification of the decisive forms of stability loss of steel structures, both at the level of a single member and for the entire structure, is therefore extremely important. In this paper, by using geometrically non-linear analysis, both the equilibrium path and the location of the singular points of the solution are determined.

Construction failures resulting in the damage of load-bearing structures made of steel members are one of the most severe, not only in terms of material loss, but also because of potential life and health loss. According to PN-EN guidelines [N1-N4], reliability evaluation of structures should rely on an idealized concept of limit states and their verification by the semi-probabilistic partial safety factor method. The role of partial safety factors is to ensure the required level of structural reliability. Probabilistic approximation (FORM [2,3,4,5,6], SORM [7,8,9]) or simulation (Monte Carlo, Importance Sampling [10,11,12,13,14]) are an extension of the semi-probabilistic limit state method. The probabilistic approach allows a more accurate and more realistic description of materials, geometric parameters and loads, acting on structures by specifying the type of probability distribution and its parameters. The safety assessment of the structure concerns both the basic design situation and exceptional situations, such as fires [15,16,17,18,19] or earthquakes [20,21,22]. However, engineers are reluctant to use numerical probabilistic methods, despite their complexity actually being hidden in computer programs. Additional effort is required from the program user to characterize the data with two parameters (expected value and standard deviation), instead of a single parameter as required in deterministic methods. It is thus necessary to provide engineers with algorithms for structural analysis with random factors considered. Explicit reliability methods lose their functionality with the increasing complexity of practical design. In response to the above, this project proposes using the interface between reliability analysis methods and numerical methods such as the finite element method for structural calculations. This approach allows building a more realistic mathematical model and, for this reason, it should be disseminated.

According to the prevailing design practice, a building structure should not only be safe but also optimized. A design engineer decides whether the response of the structure is satisfactory, and that response is dependent on the assumptions and requirements made. Optimal designs are often very sensitive to a random scatter of model parameters and external actions. Solutions that perform well for nominal values may prove unacceptable once the parameter randomness is taken into account. It therefore seems natural to extend the formulation of deterministic optimization with the random scatter of parameter values. Such a formulation is offered by robust and reliability optimization methods [23,24,25]. However, effective use of such tools requires improvements in stochastic analysis methods and development of appropriate engineering software. The following paper is an attempt to fill this gap.

## 2. Materials and Methods

The proposal to optimize the steel roof frame, taking into account the random nature of the design parameters, is the main goal of this paper. Structures of this type are characterized by a strongly non-linear response. In this situation, conducting a linear static-strength analysis is insufficient. In this paper, a geometrically nonlinear analysis in an incremental formulation is used for the calculations. After dimensioning the structure, the equilibrium path was determined together with the location of the boundary point. Based on the coordinates of the boundary point, the form of the limit state function was defined as a condition of not exceeding the permissible displacement. The reliability index was calculated using first-order reliability method. In the final phase, the roof framing was calculated using two different formulations of the optimization problem along with a re-evaluation of the safety level by calculating the reliability index. This paper adopts deterministic optimization as a reference point. Next, by combining Abaqus [26] and Numpress software [27,28], the robust design optimization was calculated. A comparative analysis of the results obtained from deterministic and robust optimization approaches allows us to present the strengths and weaknesses of both methods for optimal structural design.

### 2.1. Geometrically Nonlinear Static-Strength Analysis

The phenomena related to stability may be decisive in the design of steel roof framing. Stability theory is distinguished in structural mechanics by the need to depart from the stiffness principle, which means that nonlinear equations of at least the second order should be used. Geometrically, nonlinear analysis (GNA) allows for a complete description of the potential form of stability loss. This type of analysis can provide the equilibrium path and information about the post-critical behavior of the structure The mathematical model of nonlinear discrete systems formulated on the basis of FEM corresponds to a system of nonlinear algebraic equations. The set of equations can be formulated in a total or in an incremental form. In the total form, equations have the form (1):

(1) $$K_{s}\left(q\right)q=P$$

where: **K_s_**—secant stiffness matrix of the structure, **q**—vector of displacement, **P**—vector of nodal load.

In the incremental form, equations have the form (2):

(2) $$K_{T}(q)\Delta q=\Delta \mu P\;+R$$

where: **K_T_**(**q**)—tangent stiffness matrix of the structure; μ−load multiplier, **R** = **P**-**F**—vector of residual forces; **F**—vector of internal forces.

The matrix **K_T_** (3) of the structure is a result of the assembly of the tangent stiffness matrices of the elements $K_{T}^{e}$:

(3) $$K_{T}=\sum_{i=1}^{e}K_{T}^{e}=\sum_{i=1}^{e}{(K}_{L}^{e}\;{+K}_{G}^{e}\;{+K}_{u1}^{e}{+K}_{u2}^{e})$$

where: $K_{T}^{e}$ is the tangent stiffness matrix of the element composed of linear stiffness matrix $K_{L}^{e}$, geometric stiffness matrix $K_{G}^{e},$ and nonlinear stiffness matrices: $K_{u1}^{e}$ and $K_{u2}^{e}$.

Methods for solving nonlinear systems of equations are often based on the Newton–Raphson algorithm, in which points lying on the equilibrium path are determined in successive steps. In each step of the algorithm, a sequence of iterations is performed in such a way that, at the end of the step, the solution is obtained with the accuracy of the given error. Depending on the method, we can distinguish to control load, displacement or arc length parameter. The basic problem in numerical analysis of nonlinear problems is the occurrence of singular points on the equilibrium path (bifurcation points, limit points, turning points). At these points, standard algorithms for solving systems of linear equations fail. In this paper, we intend to use the current stiffness parameter method [29,30] and the constant arc length method [31,32] to determine and evaluate equilibrium paths.

The current stiffness parameter (CSP) is the ratio between the scaled quadratic forms of the incremental stiffness in initial and current steps (4), respectively.

(4) $$\mathrm{CSP}=\frac{{\Delta q}^{0T}\cdot K_{T}^{0}\cdot {\Delta q}^{0}}{{\Delta q}^{\mathrm{iT}}\cdot K_{T}^{i}\cdot {\Delta q}^{i}}$$

It is a measure of changes in stiffness matrix **K_T_** of the system during motion in N-dimensional displacement space of solutions. The current stiffness parameter can have many different applications:

- estimation of the system stiffness by a changing value,
- estimation of stability of the investigated segment of an equilibrium path by checking the changing sign,
- selection of effective step length,
- control near limit points.

**Figure 1:**
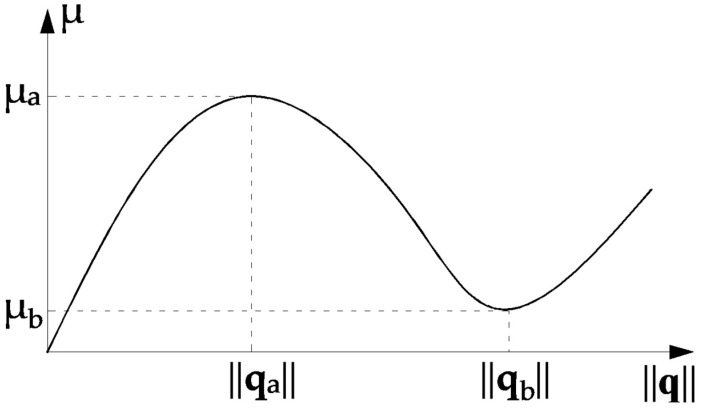
Dependence of load parameter *μ* on the norm ||**q**||.

**Figure 2:**
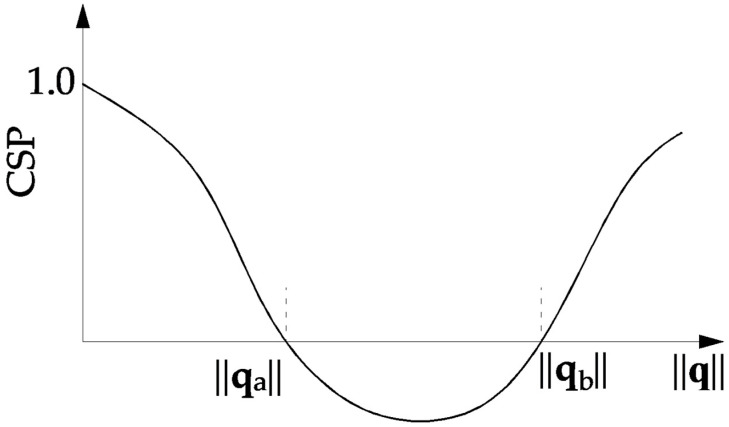
Dependence of CSP parameter on the norm ||**q**||.

Figure 1 shows a typical snap-through problem (load parameters *µ* versus some norm of displacement vector ||**q**||). The associated curve for CSP as a function of ||**q**|| is traced in Figure 2. It is seen that at the extreme points of the load–displacement curve CSP has the value zero. In this situation, the incremental stiffness matrix **K_T_** is singular. CSP is positive for the stable branches of the load–displacement curve. The instable configurations are characterized by negative values of CSP. The current stiffness parameter may actively be used in the selection of effective step length. The basic idea is that the change in CSP should be close to the same for all load steps. This implies that the incremental stiffness should be allowed to change by a prescribed magnitude for each new step. 

**Figure 3:**
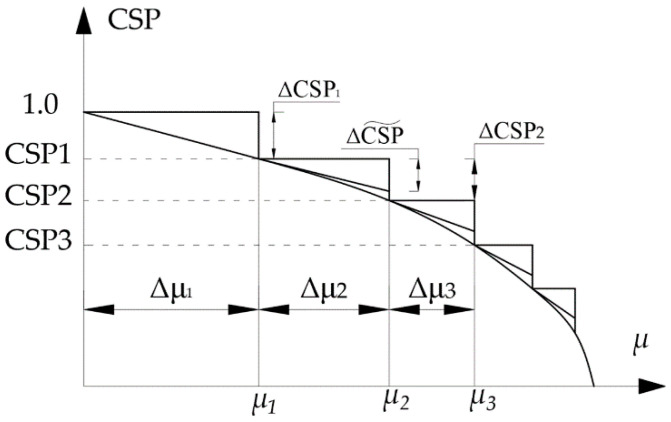
Estimation of effective step length.

Figure 3 gives an illustration of the process. The anticipated change in CSP per step is denoted as ΔCSP. This quantity is given as an input to the computer program.

**Figure 4:**
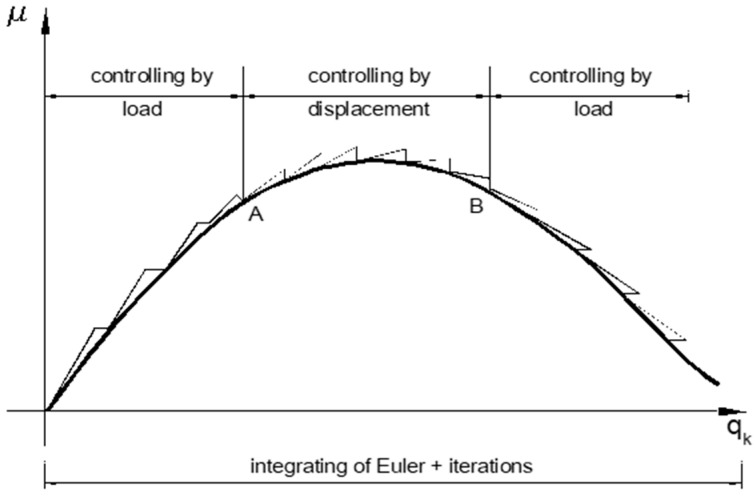
Control of iteration around extreme points.

The current stiffness parameter may actively be used in the controlling of iteration around extreme points (Figure 4).

### 2.2. First-Order Reliability Method

Traditionally, ensuring the security of an engineering system is realized by means of safety factors. Structural reliability offers an alternative approach to assess the safety of an engineering system. In structural reliability, uncertainties in loading, material properties and geometry are taken into account, explicitly. In structural reliability, the failure event F is defined in terms of a so-called limit function g(**X**), where **X** is the vector of random variables of the problem. We can determine the probability of failure using the integral (5):

P_f_ = ∫_Ωf_ f(**x**)d**x** (5)

where f(**x**) = f(x_1_, x_2_, …, x_n_), the joint probability density function, Ωf—failure area (g(**X**) ≤ 0).

**Figure 5:**
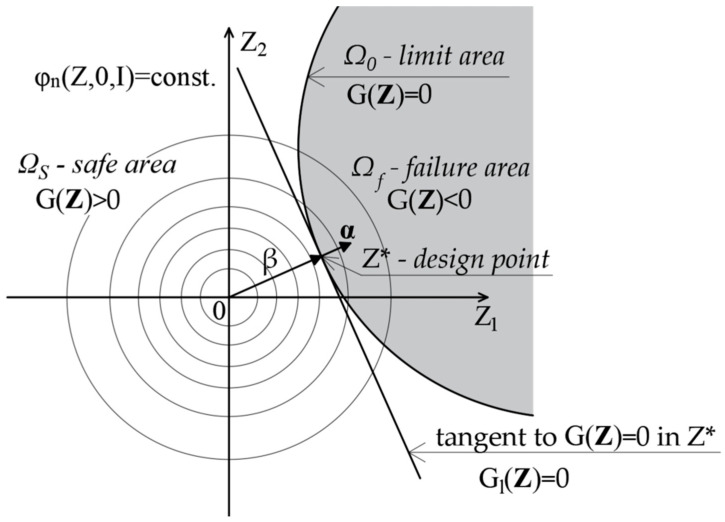
The first-order approximation of the failure probability value.

In the general case, when the distribution of the vector of base variables **X** is not a vector with a Gaussian distribution, the random variables are transformed to the standard Gaussian space **Z**. Now, the problem of reliability analysis is formulated using the limit function G(**Z**). After the transformation of the random variables **X** into the Gaussian standard space **Z**, the linearization of the limit function is performed by expanding the limit function into a Taylor series at a point lying on the limit surface, called the design point (Z* in Figure 5). This point is the point closest to the origin of the coordinate system. Due to the properties of the Gaussian standard space, the value of the joint probability density function corresponding to the failure at this point is the largest. It is the most likely point of failure of all points on that surface. If there is a failure, it is most likely at this point. The value of the reliability index obviously depends on the correct determination of the design point. This problem can be solved efficiently with the aid of gradient optimization algorithms that minimize the distance of the design point on the curve from the center of the coordinate system (Figure 5). In the FORM method the hyperplane is described by the Equation (6):

G_l_ (**Z**) = −**α^T^**∙**Z** + β (6)

where α-unit vector is anti-gradient at the design point, the β-reliability index of Hasofer–Lind.

### 2.3. Robust Optimization

The design of large and complex structures makes engineers solve issues, such as building safety, execution cost minimization and weight reduction. Increasing emphasis is thus put on material use optimization methods which have become an indispensable tool in rational structural design. Software based on what is popular in structural engineering, the finite element method usually has modules to perform optimization as standard, but only in the deterministic range. In the traditional deterministic approach, the randomness of design variables and other parameters involved in the optimization formulation are accounted for by partial safety factors. Partial safety factors defined by appropriate design standards are calibrated so that they can be applied to the widest possible range of design tasks. However, this approach often leads to overly conservative solutions. Partial safety factors are not directly related to the random scatter of the design variable values, therefore, optimal designs do not automatically provide the assumed level of reliability. When an adequately high level of safety is one of the most important requirements for the designed structure, formulating the problem as a reliability-based design optimization (RBDO) problem is worth considering [33,34,35]. In the RBDO framework, design constraints are formulated using failure probabilities. The failure probabilities are understood as the probability of exceeding certain allowable ultimate or serviceability states defined by appropriate limit state functions.

Robust design optimization belongs to non-deterministic optimization formulations. Because this approach includes the effect of structural parameter randomness on the response scatter, it usually increases structural reliability [36,37]. A typical RBDO objective function usually has the terms for structural response variability. The constraints may be deterministic or may be expressed by the first two statistical moments. More attention is paid to the adequate performance of structures subjected to small parameter variations. Unlike other types of optimization (e.g., reliability-based design optimization), imprecise specification of the types of probability distributions is not significant. The values of the first statistical moments of the response depend primarily on the first moments of the random variables. In the absence of adequate data, a uniform or normal distribution of the variables is often assumed.

The robust optimization algorithm has seven steps:

1. Specify the feasible region according to congruent with (7) and (8) and select the weighting factor γ.
2. Generate N realizations of the vector of design variables uniformly spaced over the current feasible region, in accordance with the optimal Latin-hypercube design.
3. Determine statistical moments of the objective and constraint functions for each of the N realizations of vector {$X_{d}$, **µ_x_**}.
4. Construct the response surface using methods, such as kriging, directly for individual statistical moments: ${\hat{µ}}_{f},\;{\hat{\sigma }}_{f},\;{\hat{µ}}_{g_{i}},\;{\hat{\sigma }}_{g_{i}},\;{\hat{\sigma }}_{c_{k}}$.
5. Solve the deterministic optimization problem
  (7) $$\mathrm{Find}\;\mathrm{the}\;\mathrm{value}\;\mathrm{of}\;\mathrm{variables}:\;X_{d},\;{µ}_{x}$$
  (8) $$\mathrm{Minimizing}:\;{\tilde{f}}^{\mathrm{DRS}}\;=\;\frac{1-\gamma }{{µ}^{*}}{\hat{µ}}_{f}\;(X_{d},{µ}_{x})\;+\;\frac{\gamma }{\sigma^{*}}{\hat{\sigma }}_{f}\;(X_{d},{µ}_{x})$$
  Subject to constraints:
  (9) $${\hat{µ}}_{g_{i}}\;(X_{d},{µ}_{x})\;-\;{\tilde{\beta }}_{i}\;{\hat{\sigma }}_{g_{i}}\;(X_{d},{µ}_{x})\;\geq 0{,\;\;\;\;i=1,\;\ldots ,\;k}_{g},$$
  (10) $${\hat{\sigma }}_{c_{k}}\;(X_{d},{µ}_{x})\;\leq \;\sigma_{k}^{u}{,\;\;\;\;\;\;\;\;\;\;\;\;\;\;\;\;\;\;\;\;\;\;\;\;\;\;\;\;\;\;\;k=1,\;\ldots ,\;k}_{c},$$
  (11) $$\hat{X_{d}}{\;}_{j}^{l}\;\leq \;\hat{X_{d}}{\;}_{j}\;\leq \hat{{\;X}_{d}}{\;}_{j}^{u}{,\;\;\;\;\;\;\;\;\;\;\;\;\;\;\;\;\;\;\;\;\;\;\;\;\;\;\;j=1,\;\ldots ,\;n}_{d},$$
  (12) $${{\hat{µ}}_{x}}_{r}^{l}\;\leq \;{µ}_{x_{r}}\;\leq {{\hat{µ}}_{x}}_{r}^{u}{,\;\;\;\;\;\;\;\;\;\;\;\;\;\;\;\;\;\;\;\;\;\;\;\;\;\;\;\;\;\;\;\;\;r=1,\;\ldots ,\;n}_{x},$$
6. Check the condition for convergence; if it is satisfied, terminate the algorithm.
7. Shift the feasible region over the optimal point determined at step 5. Reduce the feasible region and return.

The weighting factor γ ∈ [0,1] in Formula (8) defines the importance of each criterion. Assuming that γ = 0, an optimization problem transforms into the mean value minimization problem, whereas for γ = 1 it becomes a problem of minimizing the variance of the objective function. Values µ* and σ* are normalizing constants. Normalization coefficients are determined based on the extreme values of appropriate moments obtained in step 3. Quantities $\hat{X_{d}}{\;}_{j}^{l},\;\hat{{\;X}_{d}}{\;}_{j}^{u},\;{{\hat{µ}}_{x}}_{r}^{l},\;{{\hat{µ}}_{x}}_{r}^{u},\;$ are the current boundaries of the feasible region.

The key element of the algorithm for the realization of a robust optimization problem is an effective method of estimating mean values and standard deviations of the objective and constraint functions. Techniques of approximating implicit functions of design variables using metamodels, i.e., response surface designs, were used for this purpose. Response surfaces are developed by fitting the approximating functions to the set of experimental points [38,39,40,41,42,43]

In order to find the global maximum, random search methods, and evolutionary or heuristic algorithms are very often used [42,43,44].

In Numpress Explore, the kriging algorithm uses a two-stage technique for solving the optimization problem. Initially, the starting point for the nonlinear Nelder–Mead simplex algorithm is found with a moderate number of iterations of the simulated annealing algorithm and continued until convergence is reached [45,46].

The assumption that the considered experimental data do not have any uncertainty associated with them is true in the case of most numerical simulations. Multiple repeated calculations on the same computer for the same input data and with the pseudorandom number generator, always set at the start, lead to the same results. However, there are applications when the results have some noise, so the use of interpolating response surfaces is not rational.

A very good example of such a situation is robust optimization, where the points building the experimental basis are the results of Monte Carlo simulations, e.g., statistical moments of the objective and constraint functions. Considering the finite size of the samples that are used in the simulations, the results are scattered. In some cases, when it is not possible to properly model the effect of selected random parameters on the function value, this effect is accounted for by adding a noise component to the function.

The kriging method is an interpolation method [47].

The choice of the experimental design has an extremely significant influence on the quality of the created response surface. The most recommended designs for deterministic, computer-generated experimental data include optimal Latin hypercubes in which the experimental points are distributed as uniformly as possible in the n-dimensional parameter space.

A Latin hypercube–LH, or an n-dimensional Latin square, is a design of experiment defined using a matrix **L** of N rows and n columns. Each column is a permutation of integers from 1 to N, where N is the assumed/set/fixed number of points.

A characteristic feature of Latin hypercube designs is that any two arbitrarily selected points do not have the same coordinate.

Multidimensional optimal Latin squares, or optimal Latin hypercubes (OLH), can be regarded as a special case of LH designs. In the work [48], Stocki proves that the simulation methods that use OLH are much more effective in the estimation of statistical moments of random functions than the classic ‘purely random’ simulation methods. In the OLH design, design points fill the design region uniformly, which is extremely important in the case of constructed response surfaces or random search methods.

Advantages of OLH designs include universality, i.e., once generated, optimal n-dimensional hypercubes with N points can be saved and used repeatedly for various tasks with n-random variables [49].

Finding the optimal Latin hypercube configuration for a large number of variables and points included in the planned numerical experiment is not easy and requires specialized algorithms. These include the CP algorithm [50] or genetic algorithms [51].

In both cases, the ultimate goal is to obtain the coordinates of N points in the **R**^n^ space (N realizations of an n-dimensional random vector). In the sample obtained, probability distributions of random variables must be considered.

In the optimization of Latin hypercube point arrangements, we can distinguish two types of criteria: the minimum distance criterion and the force criterion.

To discuss the criteria, it is necessary to introduce definitions of two functions.

Function d(**L**) can be defined as the squared Euclidean distance between two points on the design of experiment (13):

(13) $$d(L)=\underset{1\leq i,j\;\leq \;N,i\neq j}{\min}\;\;\|x_{i}-x_{j}\|{}^{2}$$

Let us denote the number of minimum distance occurrences as n_d_. For a given realization of the Latin hypercube, we then get n_d_(**L**).

A function G, obtained in physical analogy, is the sum of repulsive forces acting on a set of electrically-charged particles (14):

(14) $$G\left(L\right)=\overset{N}{\underset{i=1}{\sum }}\;\overset{N}{\underset{j=i+1}{\sum }}\frac{1}{\|x_{i}-{\;x}_{j}\|{}^{2}}$$

The criterion of the minimum distance is as follows:

*A Latin hypercube **L*****_1_***is considered better than **L*****_2_***if d(**L*****_1_***) > d(**L*****_2_***), whereas when d(**L*****_1_***) = d(**L*****_2_***), **L*****_1_** *is better than **L*****_2_***if n_d_(**L*****_1_***) < n_d_(**L*****_2_***). For the force criterion, **L*****_1_***is said to be better than **L*****_2_***if G(**L*****_1_***) < G(**L*****_2_***).*

**Figure 6:**
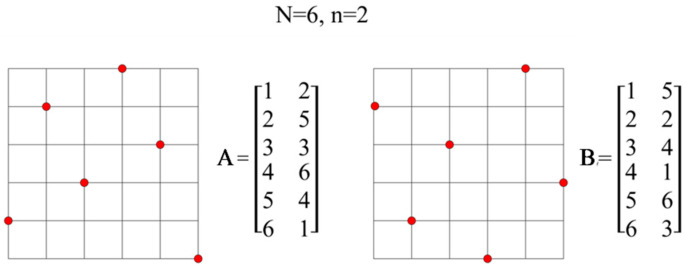
An example of Latin hypercubes.

An example (Figure 6) of assessing the quality of hypercubes according to the criteria of distance and force.

For cubes A and B, we get:

Square of the smallest (Euclidean) distance between two points on the plan of experiments (15):

(15) $$d(A)=\underset{1\;\leq \;i,\;j\;\leq \;N,\;i\;\neq \;j}{\min}\|x_{i}-{\;x}_{j}\|{}^{2}=5\;\;\;\;=\;\;\;\;\;\;d(B)=\underset{1\;\leq \;i,\;j\;\leq \;N,\;i\;\neq \;j}{\min}\|x_{i}-{\;x}_{j}\|{}^{2}=5$$

According to the criterion of the shortest distance, it is assumed that when d(L1) = d(L2), L1 is better than L2 if n_d_(L1) < n_d_(L2).

In this case, for d(**A**), the number of occurrences of the minimum distance is 5, while for d(**B**) it is 3.

According to the distance criterion, hypercube A is worse than B.

The sum of the mutual forces acting on the set of electrically charged particles (16):

(16) $$G\left(A\right)=\overset{N}{\underset{i=1}{\sum }}\;\overset{N}{\underset{j=i+1}{\sum }}\frac{1}{\|x_{i}-{\;x}_{j}\|{}^{2}}=1.67\;>\;\;G\left(B\right)=\overset{N}{\underset{i=1}{\sum }}\;\overset{N}{\underset{j=i+1}{\sum }}\frac{1}{\|x_{i}-{\;x}_{j}\|{}^{2}}=1.52$$

In the force criterion, L1 is better than L2 if G (L1) <G (L2).

According to the strength criterion, cube A is worse than B.

A value of a given criterion can be improved (by maximizing the first or minimizing the second criterion) as a result of modifying the points in the Latin hypercubes. The obtained design of experiment that uniformly fills the volume of an n-dimensional hypercube, thereby avoiding excessive concentration of the points in some areas.

Both in the case of the CP algorithm and genetic algorithms, the condition of convergence comes down to comparing the current change in the criterion value to the initial change. The implementation of the convergence condition using the example of the force criterion that uses function G (14) in the case of the CP method will be presented below.

Let ∆G_1_ be the change in the function G value after the first iteration. The convergence condition is checked after each iteration k and takes the form (17):

(17) $${\Delta G}_{K}{\;<\;\varepsilon \;\Delta G}_{1}$$

where ${\Delta G}_{K}$ is a change in the value of function G at the *k*-th iteration, ε is the chosen parameter of convergence.

Satisfying the above inequality causes the algorithm to terminate. The longer the optimization process takes, the greater the values of N and n and the smaller the value of parameter ε.

Each column of matrix **L** corresponds to one random variable having a set probability distribution. It is a permutation of numbers 1 to N. Realization x_k_(m) of the random variable $X_{K},\;1\;\leq \;k\;\leq \;n$, corresponding to m, 1 ≤ m ≤ N, in the *k*–th column of the matrix **L** is determined using the distribution of the variable X_k_ (18):

(18) $$x_{k}(m)\;=\;F_{X_{K}}^{-1}\left({\tilde{x}}_{m}\right)$$

where:

(19) $${\tilde{x}}_{m}\;=\;\frac{m}{N}\;-\;\frac{1}{2N}$$

${\tilde{x}}_{m}$ (19) is a realization of the random variable ${\tilde{x}}_{m}$ with standard uniform random distribution. $x_{K}$ is a realization of the random variable X_k_ with cumulative probability distribution $F_{X_{K}}^{-1}\left(x\right)$.

**Figure 7:**
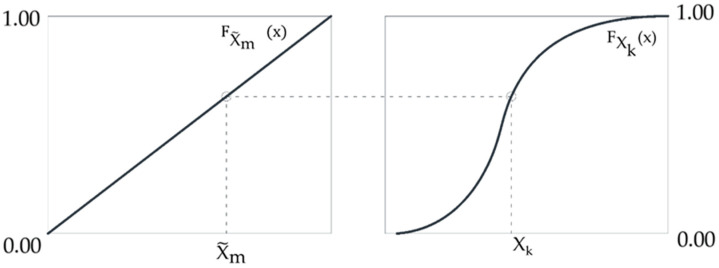
The transformation of the samples of the standard uniform random variable ${\tilde{x}}_{m}$ into samples of X_k_.

Our aim is to generate samples of the random variable X_k_ with arbitrary distribution, described by $F_{X_{K}}^{}\left(x\right)$. This is achieved by generating samples of the standard uniform random variable ${\tilde{x}}_{m}$ and transforming them into samples of X_k_ (Figure 7).

In the above method, the range of variation of each random variable is divided into N intervals of equal probability. Realizations x_k_^(i)^, I = 1, …, N, correspond to the centres of the intervals for variable X_k_. Another solution is a random choice of x_k_^(i)^ inside the *i*-th interval.

In general cases, random variables can have any probability distributions and can be correlated. The method of generating the realizations of random variables with the help of the design of experiment generated by the Latin hypercube, however, does not consider relations between variables. In order to use Latin cubes in random simulations, original variables must be transformed to a set of independent random variables. Depending on whether the functions of the total probability density or only the boundary probability distributions of random variables and the correlation coefficient matrix are known, the Rosenblatt transformation [52] or the Nataf transformation [53] can be applied. Maintaining a probability measure, both transformations convert the original random variables into independent Gaussian standard variables. The realizations of the variables generated in the standard space according to (18) are then converted to original X variables using inverse transformation. However, it should be noted that these transformations do not guarantee uniform point distribution when projected onto individual axes. The transformation approach to independent variables is universal. Since creating new Latin hypercubes for each correlation matrix is not required in this approach, the previously prepared, optimal Latin hypercube can be used for any correlation of random variables.

## 3. Results

**Figure 8:**
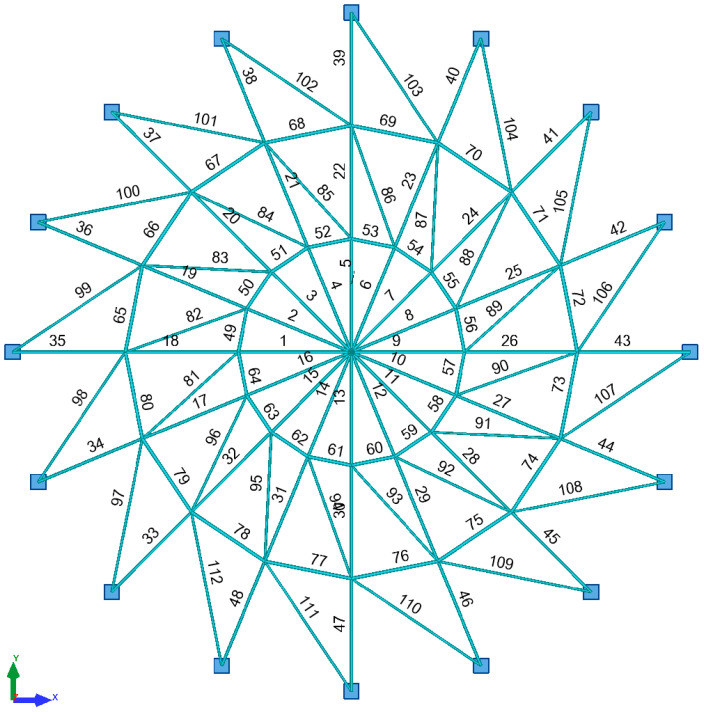
Lattice dome geometry.

A steel single-layer shallow frame roof modelled with truss elements was analyzed. A force of P = 5 kN was applied to the structure at each node. The bars were designed of S235 steel with the yield strength fy = 235 MPa, Young’s modulus E = 210 GPa and Poisson’s ratio v = 0.3 [N1]. The geometry of the considered structure is shown in Figure 8. The frame roof is supported by 16 reinforced concrete columns 8 m in height. The rigid reinforced concrete tie beam in the roof provides immovable support.

Linear static-strength analysis was performed as the first step. Table 1 summarizes the values of the cross-sectional forces, load capacity for the most stressed elements of individual bar groups and the limit values of displacements of nodes 2 to 17. Three groups of bars are distinguished in the structure in accordance with Table 2.

**Figure 9:**
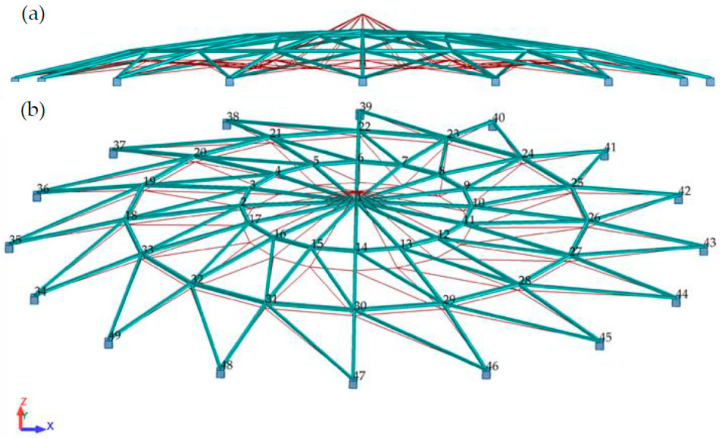
Lattice dome deformation, (**a**) side view, (**b**) 3D view.

In the second step, a linear buckling analysis was performed. Figure 9 shows the deformation of the structure. The value of the critical load factor was µ_cr_ = 1.4596.

**Figure 10:**
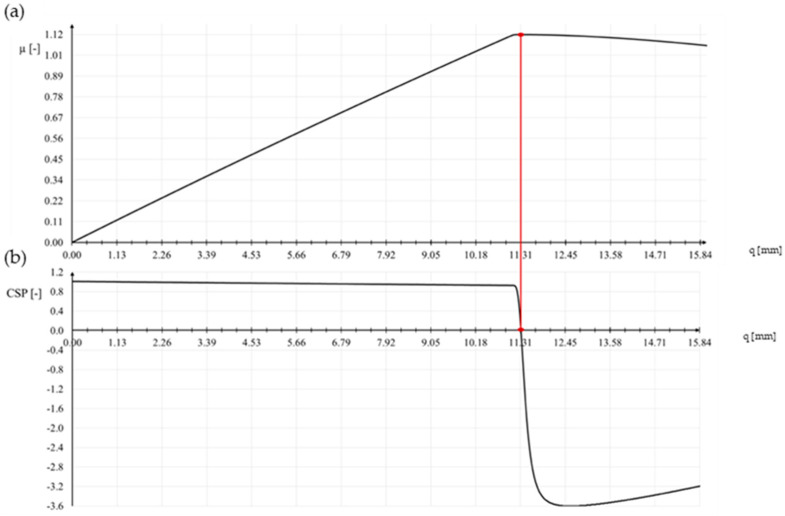
(**a**) Equilibrium path, (**b**) CSP-q relationship.

Linear analysis (LA) with linear bifurcation analysis (LBA) performs well in the design of typical high-rise steel roofs. The situation changes radically when the structure is a shallow single-layer lattice dome. Such structures are subject to large displacement gradients; therefore, it is absolutely necessary to use geometrically nonlinear analysis in the design process. The loss of stability in shallow single-layer lattice domes is related to snap-through at the nodes and not to the buckling of the members. The phenomenon is sudden and creates a domino effect. Snap-though in the position of one node leads to snap-through at subsequent nodes. It is accompanied by a sharp decrease in the stiffness of the structure. In Figure 10b, we can see that the value of the current stiffness parameter does not change significantly until snap-through occurs. However, just before it occurs, the parameter CSP virtually tends to zero vertically. The load responsible for snap-through is 5 kN × 1.12 = 5.6 kN (Figure 10a). The limit displacement is 11.31 mm (Table 3).

**Figure 11:**
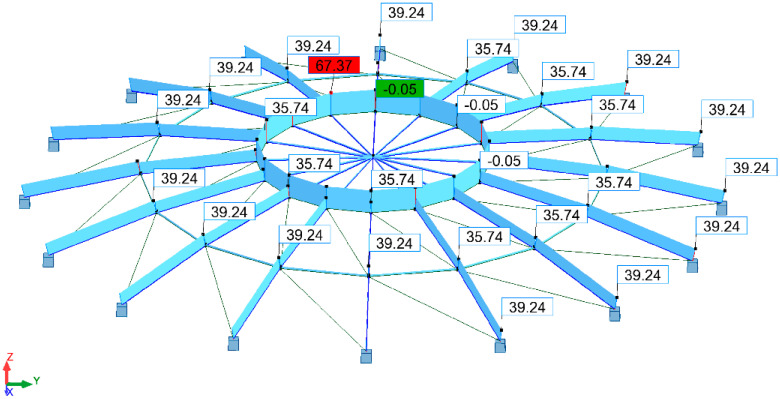
Structural stresses according to von Mises stress hypothesis [MPa].

In the final step, the stresses for individual groups of bars were verified at the moment of loading the structure with force P = 5.6 kN (Figure 11).

Internal forces and capacities for the support bars are compiled in Table 4. The parameters that are necessary for verifying the buckling of a bar are given in Table 5.

**Figure 12:**
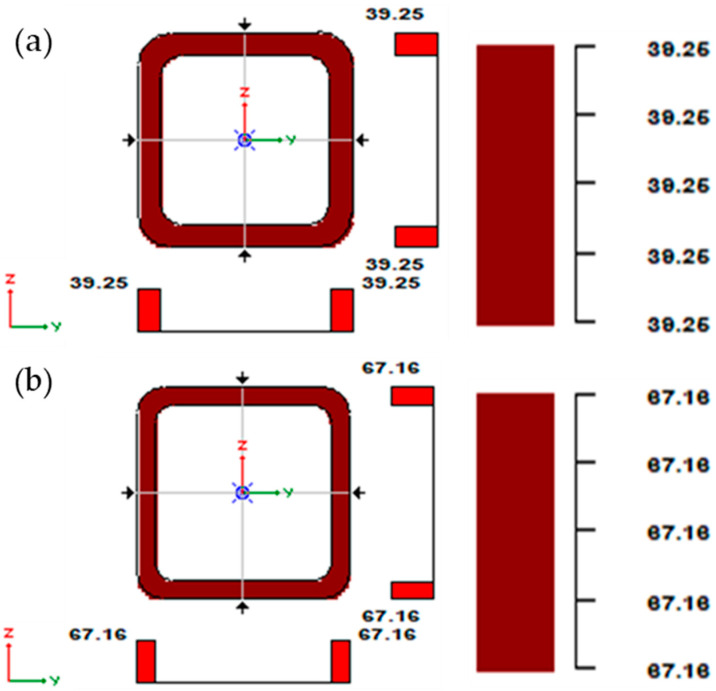
Stresses in the cross-section: (**a**) meridian, (**b**) parallel.

The stresses in bar 34, modelled from a steel pipe RK 60 × 60 × 6.3 (meridian), and in bar 59, modelled from a steel pipe RK 70 × 70 × 6 (parallel), were considered. The lengths of the elements were as follows: 2568.9 mm for the supporting meridian and 979.4 mm for the parallel. Figure 12 shows the maximum stress values in bars 34 and 59.

The stresses take a value of 39.25 MPa in the case of the support meridians and 67.16 MPa in the case of the parallels. It is worth noting that the stresses do not exceed 68 MPa, while the yield strength of S235 steel is fy = 235 MPa.

These results demonstrate that the stability of individual members is maintained. The decisive form of the loss of stability is the global loss of stability due to nodal snap-through. Therefore, for further calculations, the conditions related to the displacement of nodes are used.

### 3.1. Reliability Analysis

The reliability analysis was carried out using the FORM [2] method when the structure was loaded with a force P = 5.0 kN (load multiplier µ = 1.0). Geometric characteristics of the cross-sections were assumed as random variables: P-surface area of the meridians and R-surface area of parallels. The random variables are described in Table 6. The variables are not correlated. The mass of the modelled structure is M = 2507.386 kg. The values of the coefficients of variation were assumed at the level of 5%.

The limit function adopted based on the analysis results was related to the maximum node displacement value and had the form (20):

(20) $$f_{s}=1-\frac{w(x)}{w_{\max}}=1-\frac{w(x)}{1.131}$$

where w(**x**) is the displacement in a given calculation step, and w_max_ is the maximum displacement from nonlinear static analysis.

The value of the reliability index at the moment of loading the structure with a force P = 5.0 kN was β = 2.934, while the failure probability was p_f_ = 0.016.

### 3.2. Deterministic Optimisation

We are looking for the optimal surface areas of cross-sections of individual groups of bars: for meridians; P, and for parallels; R.

Mass of the structure will be the objective function (21):

(21) $$f_{C}=\mathrm{minimum}\;(\rho \cdot (\overset{48}{\underset{i=1}{\sum }}L_{i}\cdot P+\overset{80}{\underset{j=49}{\sum }}L_{j}\cdot R))=\min\;(\mathrm{Masa})$$

where L_i_ is the length of *i*-th bar from meridians, and L_j_ is the length of *j*-th bar from parallels.

The simple constraints described in Table 7 represent the upper and lower bounds on the sought design variables.

The inequality constraint (22) was formulated as a condition of not exceeding the permissible vertical displacement of the node w_d_ = 1.131 cm:

(22) $$g\left(x\right)=w(x)\;-{\;w}_{d}=w(x)-1.131\;<\;0$$

The deterministic optimization was carried out using the Nelder–Mead simplex method with the maximum number of iterations N = 1000 and convergence parameter ε = 1.0 × 10^−8^. The obtained dimensions of the cross-section are summarized in Table 8. The value of the objective function for this case was 2418.096 kg

The probability of failure and the reliability index, which in this case were also subject to verification, were p_f_ = 0.068 and β = 1.488, respectively.

### 3.3. Robust Optimisation

For robust optimization, random and design variables (µ_P_, µ_R_), the objective function and constraints were defined. The value of the coefficient of variation was set at 5%.

For the case under consideration, the robust optimization problem takes the form (23)–(25):

Find values of variables: µ_P_, µ_R_ (23)

Minimizing:

(24) $$f_{C}=\frac{1-\gamma }{\eta *}[\mathrm{Mass}]+\frac{\gamma }{\sigma *}\sigma \;[\mathrm{Mass}]$$

Subject to constraints:

(25) $$E\left[w(x)\;-\;1.131\right]\;-\;\tilde{\beta }\cdot \sigma \;\left[w(x)\;-\;1.131\right]\;\geq \;0\;12.455\;\leq {\;\mu }_{P}\leq \;13.755\;14.25\;\leq {\;\mu }_{R}\;\leq \;15.75$$

where α ∈ [0,1] defines the importance of each criterion, η*, σ* normalizes constants, and w(**x**) − 1.131 allows vertical displacement constraint.

In order to confirm the correctness of the performed calculations, two methods of response surface construction were used: kriging and the second-order method. Parameters were assumed as follows: γ = 0.5 and $\tilde{\beta }$ = 2.0. The design variable values obtained from both methods are compiled in Table 9.

In the robust optimization algorithm used in Numpress Explore, the optimal point obtained as the minimization of the function approximating the objective function is verified by means of validation procedure. In the validation process, we confirm the correctness of the obtained results by solving the problem (substituting the values of decision variables) for the original objective function (FEM analysis). During the analysis, a set of points is created from which the smallest value of the objective function is verified. In a properly designed analysis, the values obtained as the optimal point are consistent with the validation results.

The probability of failure and the reliability index, which in this case were also subject to verification, were p_f_ = 1.84 × 10^−3^ and β = 2.089, respectively.

## 4. Discussion

According to the current design trend, the structure should not only be safe, but also optimal. FEM-based programs are equipped with basic optimization modules, but only in a deterministic version. The random deviations of the design parameters clearly show the shortcomings of this analysis. The initial mass of the structure was 2507.386 kg. The decisive global form of loss of stability was related to the nodal snap-through. Therefore, for further calculations, the conditions related to the displacement of nodes were used. On this basis, the limit function was formulated. The value of the reliability index was 2.934, while the failure probability was 0.016. Deterministic optimization definitely “slimmed down” the structure, but at the cost of its safety. The mass of the structure was 2418.096 kg, while the reliability index decreased to 1.488. As a result of robust optimization, the cross-sectional area of individual groups of bars and the mass of the structure were modified. In this case, an increase in the value of the reliability index and a decrease in the probability of failure in relation to deterministic optimization were observed. These values were: β = 2.089 and pf = 1.84 × 10^−3^, respectively. The mass of the structure was 2435.780 kg. Additionally, in order to verify the calculations, two different methods of constructing the response surface were used: kriging and second-order method. Based on the results presented in Table 9, we can conclude that the analysis was carried out correctly.

## 5. Conclusions

1. Shallow steel roof framing characterizes strong nonlinear effects. Therefore, calculations should be based on a geometrically nonlinear analysis. The buckling of individual members does not always lead to the loss of stability of the structure. The phenomenon of the snap-through is often the decisive form of loss of stability.
2. Optimal designs are usually particularly sensitive to parameter imperfections. Optimal solutions located on the border of the acceptable area may, due to imperfection, enter the hazardous area relatively easily, and thus turn out to be completely useless if the parameter values differ even slightly from the assumed nominal values.
3. An indispensable element of structure design should be the support of deterministic optimization with robust optimization. As a result of robust optimization, we obtain a structure that is less optimal (with a negligibly greater mass), but definitely safer, as evidenced by the values of reliability indexes. Taking into account the uncertainty of the design parameters in the formulation of the robust optimization unequivocally solves this problem, giving the designer control over the safety level of the structure.

## Figures and Tables

**Table 1:** Values of cross-sectional forces and load capacities for the most stressed structure elements and values of maximum vertical and horizontal displacements for nodes 2–17 in linear analysis.

Internal Force/Load Capacity	Meridian Bar No 33	Parallel Bar No 56
N_ed_ [kN]–axial force	45.675	85.187
N_c,Rd_ [kN]–design capacity of the cross-section at uniform compression	307.850	352.500
N_b,Rd_ [kN]–design buckling capacity of the element in compression	151.618	335.679
Maximum vertical displacement [mm]	9.36	
Allowable vertical displacement [mm]–D/300	50.10	
Maximum horizontal displacement [mm]	1.08	
Allowable horizontal displacement [mm]–H/150	6.80	

**Table 2:** Cross-sections of individual bar groups and the maximum bar stress.

Group	Node No.	Profile	Stress
Meridian	1 to 48	RK60 × 60 × 6.3	30%
Parallel	49 to 80	RK70 × 70 × 6	25%
Diagonals	81 to 112	RK50 × 50 × 5	0%

**Table 3:** Values of cross-sectional forces for the most stressed elements in the structure and values of maximum vertical and horizontal displacements for nodes 2–9 for the critical load in geometrically nonlinear analysis.

Internal Force/Load Capacity	Meridian Bar No. 34	Parallel Bar No. 59
Ned [kN]–axial force	51.174	100.564
Stress [%]	34	30
Maximum vertical displacement [mm]	11.31	
Allowable vertical displacement [mm]–D/300	50.1	
Maximum horizontal displacement [mm]	1.32	
Allowable horizontal displacement [mm]–H/150	6.80	

**Table 4:** Internal forces and cross-section capacities.

Internal Force/Load Capacity	Meridian Bar No. 34	Parallel Bar No. 59
N_Ed_ [kN]	51.174	100.564
N_c,Rd_ [kN]	307.850	352.500
N_b,Rd_ [kN]	151.618	335.679

**Table 5:** Buckling parameters.

Parameter	Meridian Bar No. 34	Parallel Bar No. 59
L_y_= L_z_—length of element [mm]	2568.88	979.44
L_cr,y_ = L_cr,z_—buckling effective length [mm]	2568.88	979.44
L_amy_ = L_amz_—slenderness of bar	118.46	37.75
L_am,y_ = L_am,z_—relative slenderness of bar	1.26	0.40
χ_y_ = χ_z_—buckling coefficient	0.49	0.95

**Table 6:** Description of random variables.

Random Variables X_i_	Mean Values [cm^2^]	Standard Deviation [cm^2^]	Coefficient of Variation [%]
P	13.1	0.655	5
R	10.7	0.535	5

**Table 7:** Design variable bounds.

Design Variable	Lower Bound [cm^2^]	Upper Bound [cm^2^]
P	12.455	13.755
R	14.25	15.75

**Table 8:** Design variable values from deterministic optimization.

Design Variable	Optimal Value [cm^2^]
P	12.456
R	14.250

**Table 9:** Comparison of the results of kriging and the second-order method.

	Kriging	Second-Order
Parameter γ	0.5	0.5
Assessment of the dispersion of the sample size	48	48
Sample size to generate RS base points	48	48
Optimal point		
Random variable P	12.455	12.455
Random variable R	14.726	14.732
Value of optimised objective function	0.971	0.972
Approximated mass of the structure	2435.53	2435.78
Approximated value of mass standard deviation	68.29	68.29
Validation		
Random variable P	12.455	12.455
Random variable R	14.726	14.730
Approximated mass of the structure	2435.53	2435.78
Approximated value of mass standard deviation	68.29	68.29

## List of Standards

N1. EN 1993-1-1. Eurocode 3: Design of steel structures–Part 1–1: General rules and rules for buildings.
N2. EN 1991-1-3. Eurocode 1: Actions on structures–Part 1–3: General actions–Snow loads.
N3. EN 1991-1-4. Eurocode 1: Actions on structures–Part 1–4: General actions–Wind actions.
N4. EN 1990: 2002. Eurocode–Basis for structural design.
